# Functional Dissection of the *Clostridium botulinum* Type B Hemagglutinin Complex: Identification of the Carbohydrate and E-Cadherin Binding Sites

**DOI:** 10.1371/journal.pone.0111170

**Published:** 2014-10-23

**Authors:** Yo Sugawara, Masahiro Yutani, Sho Amatsu, Takuhiro Matsumura, Yukako Fujinaga

**Affiliations:** Laboratory of Infection Cell Biology, International Research Center for Infectious Diseases, Research Institute for Microbial Diseases, Osaka University, Yamada-oka, Suita, Osaka, Japan; University of Wisconsin, Food Research Institute, United States of America

## Abstract

Botulinum neurotoxin (BoNT) inhibits neurotransmitter release in motor nerve endings, causing botulism, a condition often resulting from ingestion of the toxin or toxin-producing bacteria. BoNTs are always produced as large protein complexes by associating with a non-toxic protein, non-toxic non-hemagglutinin (NTNH), and some toxin complexes contain another non-toxic protein, hemagglutinin (HA), in addition to NTNH. These accessory proteins are known to increase the oral toxicity of the toxin dramatically. NTNH has a protective role against the harsh conditions in the digestive tract, while HA is considered to facilitate intestinal absorption of the toxin by intestinal binding and disruption of the epithelial barrier. Two specific activities of HA, carbohydrate and E-cadherin binding, appear to be involved in these processes; however, the exact roles of these activities in the pathogenesis of botulism remain unclear. The toxin is conventionally divided into seven serotypes, designated A through G. In this study, we identified the amino acid residues critical for carbohydrate and E-cadherin binding in serotype B HA. We constructed mutants defective in each of these two activities and examined the relationship of these activities using an *in vitro* intestinal cell culture model. Our results show that the carbohydrate and E-cadherin binding activities are functionally and structurally independent. Carbohydrate binding potentiates the epithelial barrier-disrupting activity by enhancing cell surface binding, while E-cadherin binding is essential for the barrier disruption.

## Introduction

Botulinum neurotoxin (BoNT) is the etiological agent of the disease botulism. Different types of this toxin are produced by various strains of the spore-forming anaerobic bacteria *Clostridium botulinum, C. butyricum,* and *C. barati*, and are conventionally classified into seven serotypes, designated A through G [1]. Recently, a novel serotype H was reported, although it is still controversial whether this designation is appropriate [2], [3]. BoNTs exist as large protein complexes by associating with non-toxic proteins termed neurotoxin-associated proteins (NAPs), which consist of non-toxic non-hemagglutinin (NTNH) and hemagglutinin (HA) [4]. BoNT and NTNH form a protein complex termed 12S toxin or M toxin. In serotypes A–D and G, another complex termed 16S toxin or L toxin is also formed, which consists of BoNT, NTNH, and HA. Type A toxin is reported to form 19S toxin, which is considered to be a dimer of 16S toxin [5].

BoNT complex is a unique toxin in that it can give rise to food-borne disease, although the target site is nerve endings. Orally ingested toxin withstands the harsh conditions of the gastrointestinal tract, enters the general circulation through the intestinal epithelial barrier, and reaches peripheral nerve endings where BoNT cleaves SNARE proteins, resulting in the inhibition of neurotransmitter release [6], [7]. NAPs are known to potentiate oral toxicity of the toxin complex. It was shown that the larger the molecular size of the toxin complex, the higher the oral toxicity [8]. NTNH stabilizes BoNT in the harsh conditions in the digestive tract, thereby potentiating the oral toxicity. HA is also reported to contribute to the stability of BoNT [9]. However, a direct association between BoNT and HA was not observed in a structural model of 16S toxin, and it remains controversial whether HA contributes to the stability of BoNT [10]. Besides the protection by NAPs, two specific activities of HA appear to enhance the oral toxicity: carbohydrate binding and the epithelial barrier disruption. It has been observed that the toxin complex containing HA interacts with epithelial cells through carbohydrate binding [11], [12]. In addition, mice that orally ingested with the toxin complex were protected by the inclusion of some kinds of monosaccharide that are recognized by HA [13]. These observations indicate that carbohydrate binding activity greatly contributes to the oral toxicity of BoNT. Meanwhile, we recently found that HA disrupts the epithelial barrier [14]. Upon the addition of HA, the barrier function of the epithelial cell monolayer, which is mediated by tight junctions, is compromised, and an influx of macromolecules through the gaps between the cells is promoted. We identified E-cadherin, a major constituent of adherens junction, as the target molecule of type A and B HAs [15]. These HAs directly bind to E-cadherin and inhibit its function, thereby disrupting tight junctions. We hypothesized that HA disrupts the epithelial barrier and allows further influx of the toxin complex that resides on the intestinal lumen, which, in turn, potentiates the oral toxicity of the toxin [16].

HA consists of three proteins termed HA1, HA2, and HA3. Alternatively, they are also referred to as HA33, HA17, and HA70, respectively. Two HA1, one HA2, and one HA3 molecules constitute the HA monomers, which trimerize via HA3 to form a whole HA complex; twelve HA molecules constitute the large whole HA complex [13], [17], [18]. Among three HA proteins, HA1 and HA3 show carbohydrate-binding activity. HA1 binds to galactose in types A through C, while HA3 proteins of these serotypes interact with sialylated molecules [12], [19], [20], [21]. Type C HA1 has an additional binding site for sialic acid [22]. Meanwhile, the E-cadherin binding site is assumed to be located in the HA2-HA3 connecting region [18]. Here, we identified amino acid residues critical for carbohydrate binding and E-cadherin binding in type B HA. Then, we assessed how these two activities are related.

## Materials and Methods

### Plasmid construction

DNA fragments encoding botulinum hemagglutinins were amplified by PCR from genomic DNA isolated from *C. botulinum* type B strain Okra or type C strain Stockholm using gene-specific primers containing restriction sites at their 5′ ends. The type B HA1 and HA2 fragments were inserted into the HindIII-KpnI site of the pT7-FLAG-1 vector (Sigma Aldrich). The type C HA1 fragment was inserted into the NotI-BglII site of the pT7-FLAG-1 vector. The HA3 fragments were inserted into the KpnI-SalI site of the pET52b(+) expression vector (Merck Millipore). The type B HA1 and HA3 fragments were also inserted into the BamHI-SalI site of the pGEX-5X-3 vector (GE Healthcare). Site-directed mutagenesis was performed using the PrimeSTAR Max polymerase (Takara Bio). The inserted regions of these vectors were confirmed by DNA sequencing.

### Protein expression and purification

Protein expression was conducted as previously described using Overnight Express Autoinduction System 1 (Merck Millipore) [18]. FLAG-tagged proteins, FLAG-HA1 and FLAG-HA2, were purified using anti-FLAG M2 agarose (Sigma Aldrich). HA3 proteins were expressed and purified as the uncleaved forms, although they are cleaved to yield two fragments, designated HA3a and HA3b (alternatively termed HA20 and HA50, respectively), in native toxin complex [4]. We previously showed that the barrier-disrupting activity of recombinant HA complex is comparable to that of native HA complex, indicating that the cleavage of HA3 is dispensable for the activity [18]. The Strep-HA3 proteins were purified using StrepTrap HP (GE Healthcare) according to the manufacturer’s instructions. GST-fused proteins, GST-HA1 and GST-HA3, were purified using GSTrap HP (GE Healthcare). For reconstitution of the HA complex, FLAG-HA1, FLAG-HA2, and Strep-HA3 proteins were mixed at a molar ratio of 4∶4:1 in PBS, pH 7.4, and incubated for 3 h at 37°C. The HA complex was captured with a StrepTrap HP followed by a brief wash with PBS, and then eluted with PBS containing 3 mM D-desthiobiotin (Merck Millipore). Purified proteins were dialyzed against PBS, pH 7.4. E-cadherin ectodomain protein was expressed in HEK293 cells, and purified as previously described [15]. Protein concentrations were determined using the BCA Protein Assay Reagent (Thermo Scientific).

### Mucin binding assay

Mucin binding assay was performed as described by Nakamura et al. with some modifications [22]. The wells of 96-well microtiter plates were coated with 10 µg of bovine submaxillary mucin (BSM) or 1 µg of porcine gastric mucin (PGM) (Sigma Aldrich). After washing with PBS containing 0.2% Tween20 and blocking with 1% BSA, mucin-coated wells were incubated with 1 µM GST-tagged HA1, HA3, or 50 nM HA complex. For the removal of sialic acid, the wells were pretreated with 5 mU/ml *Arthrobacter ureafaciens* neuraminidase (Nacalai Tesque) for 1 h at 37°C before the addition of HA. Bound proteins were probed with rabbit anti-GST antibody (Sigma Aldrich) or rabbit anti-B16S antibody [15], followed by peroxidase-labeled goat anti-rabbit IgG (Jackson Immunoresearch). The plates were developed using ABTS (Roche Diagnostics), and absorbance was measured at 405 nm using a microplate photometer (Thermo Scientific).

### Cell culture

Caco-2 cells, the human colon adenocarcinoma-derived cell line, were obtained from Riken Cell Bank, and were grown in a 5% CO_2_ humidified incubator with Eagle’s MEM (Life Technologies) supplemented with 20% heat-inactivated fetal bovine serum.

### Immunofluorescence

Cells grown on Transwell pore filters were fixed with 4% paraformaldehyde in PBS for 20 min. Cells were incubated with rabbit anti-B16S antibody before permeabilization. After permeabilization with 0.5% Triton X-100 in PBS for 5 min, monolayers were incubated with primary antibodies, mouse anti-E-cadherin (BD Biosciences) or anti-ZO-1 (Zymed) monoclonal antibodies, probed with secondary antibodies coupled to Alexa488 (Invitrogen) or Cy3 (Jackson Immunoresearch), and mounted with ProLong Antifade kit (Invitrogen). All procedures were carried out at room temperature. Images were captured with an Olympus IX71 microscope equipped with a LCPlanFl 40×/0.60 objective (Olympus) and a CSU-X1 confocal scanner unit (Yokogawa). Images were analyzed with MetaMorph imaging software (version 7.7.10.0, Universal Imaging).

### Measurement of transepithelial electrical resistance (TER)

Measurement of TER was carried out using a Millicell-ERS (Merck Millipore) as described previously [23].

### HA pull-down assay

The concentration of the E-cadherin ectodomain protein was adjusted to 100 nM in Hepes buffer (20 mM Hepes-NaOH, pH 7.35, 2 mM CaCl_2_, 150 mM NaCl, and 0.01% Triton X-100). A 200 µl aliquot of E-cadherin solution was mixed with StrepTactin Superflow agarose gel (Merck Millipore) coupled with HA proteins, and rotated for 1 h at 4°C. The gels were washed twice with 1 ml of Hepes buffer and bound proteins were eluted with SDS-PAGE sample buffer. Samples were separated by SDS-PAGE and transferred to a PVDF membrane. Western blot was performed with an anti-mouse E-cadherin antibody, ECCD-2 (Takara Bio). HA proteins were stained with Coomassie blue to show that comparable amounts of HA proteins were coupled to the affinity resin among the compared samples.

## Results

**Figure 1:**
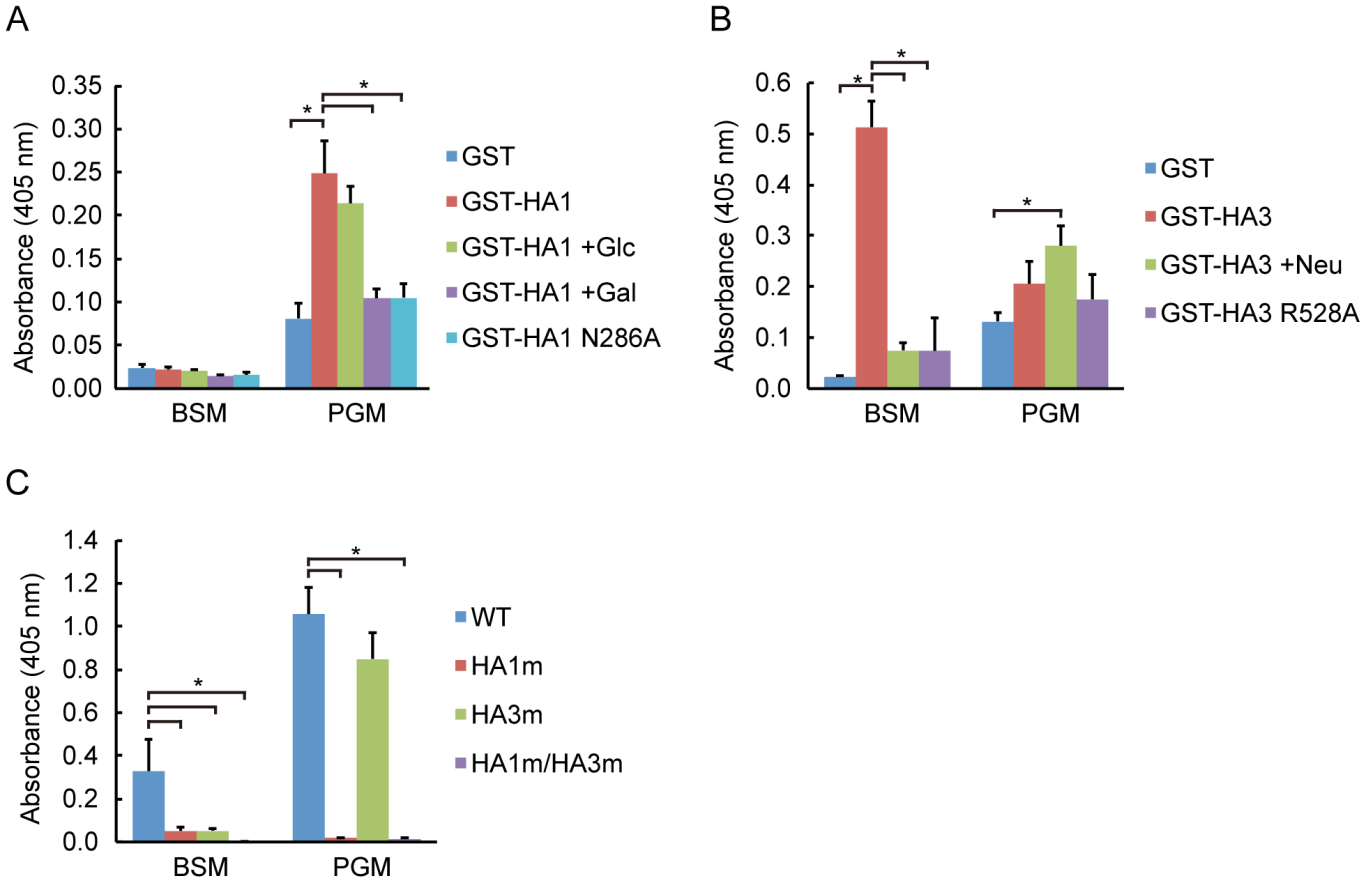
HA/B complex binds to mucins through carbohydrate recognition by HA1/B and HA3/B. (A) Mucin binding of GST-HA1/B. Mucin binding of GST, GST-HA1/B, and GST-HA1/B harboring Asn286 to Ala mutation (N286A) was assessed as described in Materials and Methods. Binding of GST-HA1/B was measured in the presence or absence of 100 mM glucose (Glc) or galactose (Gal). HA1/B preferentially bound to PGM in a galactose-binding-dependent manner. (B) Mucin binding of GST, GST-HA3/B, and GST-HA3/B harboring Arg528 to Ala mutation (R528A). Binding of GST-HA3/B was assessed with the coated mucin pretreated with (+Neu) or without 5 mU/ml neuraminidase. HA3/B preferentially bound to BSM in a sialic-acid-dependent manner. (C) Mucin binding of HA/B complex (WT) and those harboring either HA1/B N286A or HA3/B R528A, or both (HA/B harboring HA1 N286A mutation, HA1 m; HA/B harboring HA3 R528A mutation, HA3 m; HA/B harboring HA1N286A and HA3 R528A mutations, HA1 m/HA3 m). The results show that carbohydrate binding activities of HA1 and HA3 are fully functional in the HA/B complex. Values are means ± S.E.M. from three independent experiments. *p<0.05, unpaired *t* test.

The galactose binding site of HA1 and the sialic acid binding site of HA3 have already been identified in serotypes A and C (HA/A and HA/C) [13], [20], [24]. The amino acid residues involved in binding are conserved among types A, B and C, suggesting that HA1/B and HA3/B could recognize galactose and sialic acid, respectively, in a similar manner to types A and C. A recent study, in which the crystal structure of HA1/B bound with its ligand lactose was solved, supports this assumption as to HA1 [25]. It was reported that the galactose binding of HA1/C is abolished by alanine substitution of Asn278, and sialic acid binding of HA3/A is abrogated by alanine substitution of Arg528 [13], [20]. We constructed mutants of HA1/B and HA3/B in which amino acids equivalent to these residues, Asn286 in HA1/B and Arg528 in HA3, were substituted with alanine (HA1 N286A and HA3 R528A). We assessed the effects of these mutations on carbohydrate binding by mucin binding assay [22], [26]. As shown in Fig. 1A, HA1/B bound to porcine gastric mucin (PGM), whose carbohydrate moiety is devoid of sialic acid, but not to bovine submaxillary mucin (BSM), which is rich in sialic acid. This binding was inhibited in the presence of galactose, showing that the binding is dependent on the galactose binding of HA1/B. HA1 N286A did not bind to PGM, indicating that Asn286 is critically involved in carbohydrate binding, as observed in other serotypes. In contrast to HA1/B, HA3/B preferentially bound to BSM (Fig. 1B). This binding was significantly decreased when the coated mucin was pretreated with neuraminidase, indicating that the binding is dependent on sialic acid. BSM binding of HA3/B was abolished by alanine substitution of Arg528, confirming that this residue is critically involved in sialic acid binding. Then, we assessed whether these carbohydrate binding sites function in the HA complex. HA complexes containing carbohydrate-binding-defective mutations were prepared as described in the Materials and Methods section, and carbohydrate binding activity was evaluated by mucin binding assay. As shown in Fig. 1C, BSM binding was greatly inhibited by either HA1 or HA3 mutation (HA1 m, HA3 m), and completely abolished in the presence of both mutations (HA1 m/HA3 m) compared to the wild-type HA/B (WT). PGM binding was abolished when the galactose binding of HA1 was defective. Collectively, these results show that Asn286 of HA1/B and Arg528 of HA3/B are critically involved in carbohydrate binding, through galactose and sialic acid recognition, respectively, and these types of binding are fully functional in the whole HA complex.

**Figure 2:**
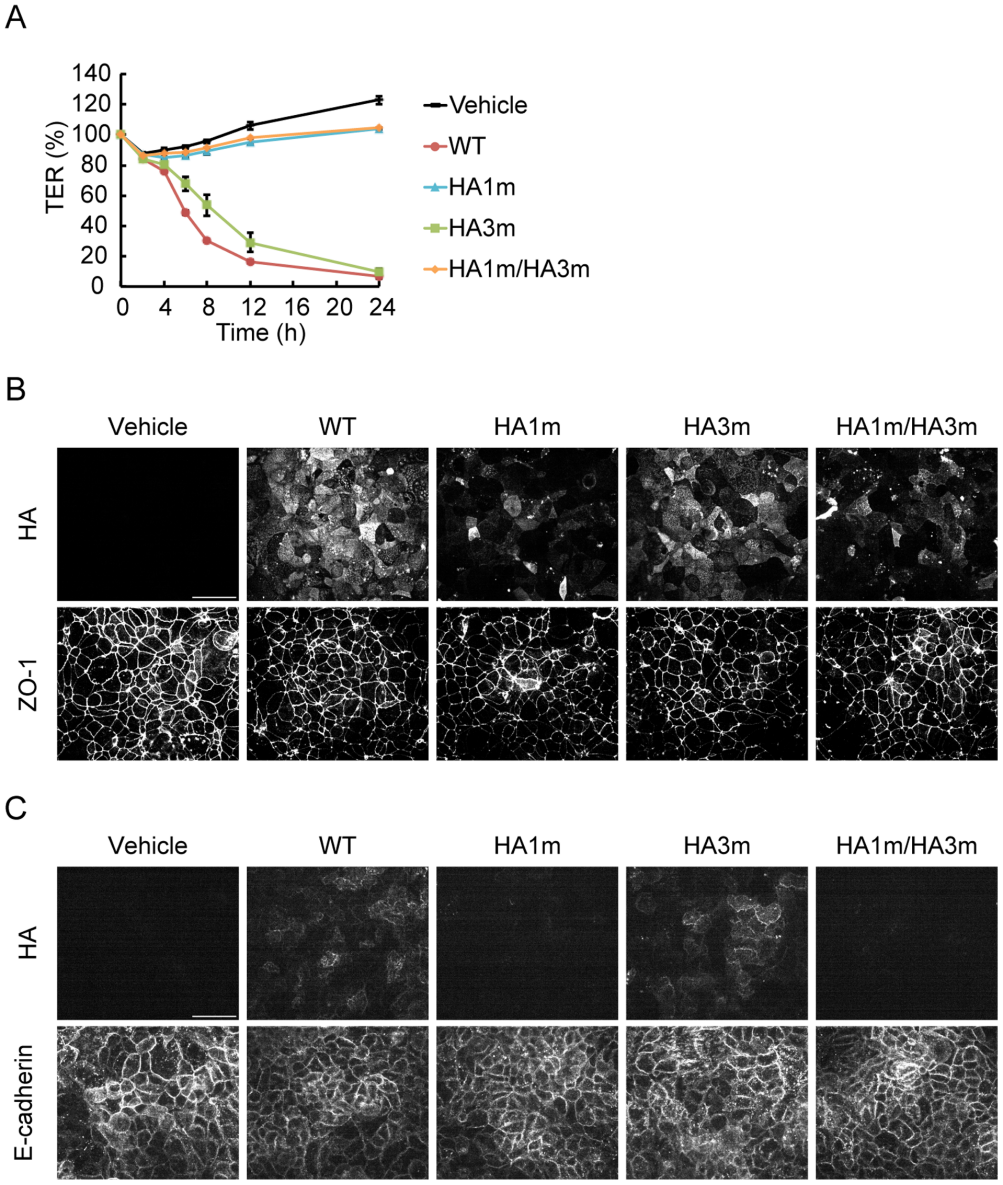
Carbohydrate binding of HA1/B is required for apical to basolateral translocation through Caco-2 cell monolayers. (A) Caco-2 cells were grown on Transwell chambers, and treated with 500 nM HA/B (WT) or those harboring carbohydrate-binding-defective mutations (HA/B harboring HA1 N286A mutation, HA1 m; HA/B harboring HA3 R528A mutation, HA3 m; HA/B harboring HA1 N286A and HA3 R528A mutations, HA1 m/HA3 m) from the upper side of the chambers. TER was measured at time points up to 24 h. The barrier-disrupting activity was inhibited by HA1 mutation. Values are means ± S.E.M. of triplicate wells. (B, C) Caco-2 cells were treated with HA the same as in A. After 1 h of treatment, cells were fixed and the apical (B) or basolateral (C) surface of the cell was stained with the anti-B16S antibody. ZO-1 and E-cadherin were also stained to show the apical and basolateral positions of the epithelial cells, respectively. HA1 mutation greatly affected the apical cell surface binding and subseqent translocation to the basolateral surface of the HA complex. Scale bars: 50 µm.

HA/B binds to E-cadherin, and in turn, disrupts the epithelial barrier [15]. We considered whether carbohydrate binding affects the epithelial-barrier-disrupting activity of HA. To address this issue, the barrier-disrupting activity of HA complex and those harboring carbohydrate-binding-defective mutations was assessed by measuring TER using Caco-2 cells. The effects of the carbohydrate binding mutations differed depending on whether the HA complex was applied from the apical or basolateral side of the cells. Since E-cadherins reside on the basolateral surface, apically applied HA needs to translocate through the epithelial cells to affect the epithelial barrier [15]. When the complex was applied from the apical side, the activity was completely inhibited by the HA1 mutation, and attenuated by the HA3 mutation (Fig. 2A). Binding to the apical surface of the cells was significantly reduced in the complex containing HA1 mutation, and apical to basolateral translocation was largely inhibited by this mutation (Fig. 2B, C). These results are consistent with our previous notion that HA1 is essential for apical to basolateral translocation and suggest that carbohydrate binding by HA1 is responsible for this activity [18].

**Figure 3:**
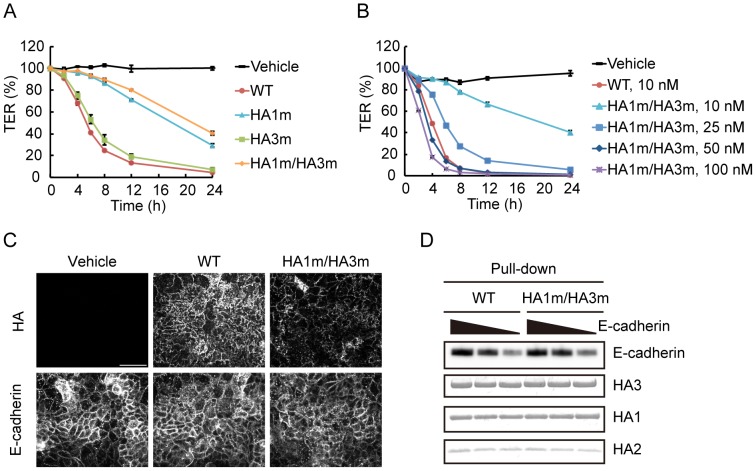
Carbohydrate binding of HA/B is not required for E-cadherin binding. (A) Caco-2 cells were grown on Transwell chambers, and treated with 10 nM HA/B (WT) or those harboring carbohydrate-binding-defective mutations (HA/B harboring HA1 N286A mutation, HA1 m; HA/B harboring HA3 R528A mutation, HA3 m; HA/B harboring HA1 N286A and HA3 R528A mutations, HA1 m/HA3 m) from the lower side of the chambers. HA1 mutation affected the barrier-disrupting activity, whereas HA3 mutation largely did not. (B) Caco-2 cell monolayers were treated with 10 nM HA/B or the indicated concentrations of HA/B carbohydrate-binding-defective mutant (HA1m/HA3m). These HAs were treated with the lower side of the chamber. Values are means ± S.E.M. of triplicate wells. The mutant showed sufficient activity to reduce the TER value completely when applied at higher concentrations (50 nM and 100 nM). (C) Caco-2 cells were treated the same as in A. After 1 h of treatment, cells were fixed and the basolateral surface of the cell was stained with the anti-B16S antibody. E-cadherin staining is also indicated. Binding to the basolateral surface of the cells was reduced in the carbohydrate-binding-defective mutant. Scale bar: 50 µm. (D) Varying concentrations of the recombinant E-cadherin ectodomain protein (300, 100, and 30 nM) was pulled-down with resin-bound HA/B complex or its carbohydrate-binding-defective mutant. The mutations did not affect E-cadherin binding.

When the complex was applied from the basolateral side, the barrier-disrupting activity was reduced but not abolished in the complex containing the HA1 mutant (Fig. 3A). Meanwhile, the activity was not largely affected by HA3 mutation. The barrier-disrupting activity of the complex harboring mutations in both HA1 and HA3 was reduced to approximately one-fifth of that of the wild type; however, it shows sufficient activity to disrupt the epithelial barrier completely when applied at higher concentrations, suggesting that carbohydrate binding is not essential for the activity (Fig. 3B, HA1 m/HA3 m 50 nM and 100 nM). Attachment of the double mutant to the basolateral surface of the cells is less efficient than that of the wild type, and its localization was relatively specific to cell-to-cell junctions (Fig. 3C). The E-cadherin extracellular domain is known to be modified with glycans, such as by N-glycosylation, O-glycosylation, and O-mannosylation [27], [28], [29]. Therefore, it is conceivable that these modifications enhance E-cadherin binding of HA by carbohydrate recognition. However, direct binding to E-cadherin was comparable between the wild type and the double mutant in a pull-down assay (Fig. 3D). These lines of evidence suggest that carbohydrate binding is not required for E-cadherin binding, but potentiates the barrier-disrupting activity by promoting cell surface attachment of HA.

**Figure 4:**
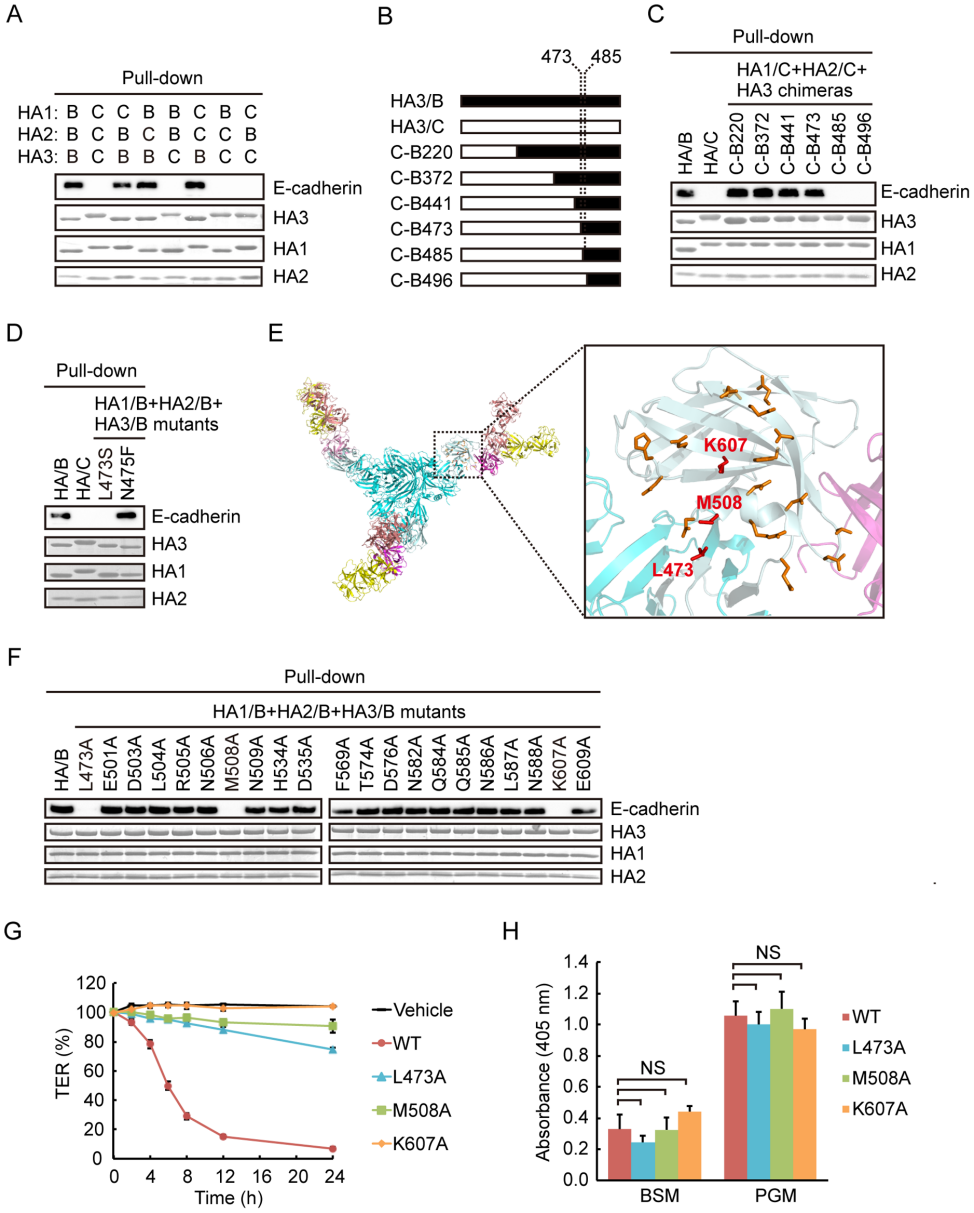
Identification of the E-cadherin binding sites in HA/B. (A) Chimeric HA complexes in which each subcomponent of the complex is replaced between types B and C were prepared, and E-cadherin binding was assessed by HA pull-down assay using the recombinant E-cadherin ectodomain protein. The results indicate that HA3/B is required for E-cadherin binding. The molecular sizes of HA proteins differ between types B and C. (B) Schematic representation of chimeric HA3 constructs between types B (black) and C (white). (C) E-cadherin binding of the HA/B, HA/C, and HA/C complexes containing each of the chimeric HA3 constructs was assessed by HA pull-down assay. (D) E-cadherin binding of the HA/B, HA/C, and HA/B complex harboring HA3 point mutations (Leu473 to Ser, L473S; Asn475 to Phe, N475F) was assessed by HA pull-down assay. E-cadherin binding was inhibited by mutating Leu473, suggesting that this amino acid residue is involved in this binding. (E) Crystal structure of the whole HA/B complex ([18], left) and a higher-magnification image of the boxed region (right). Leu473, Met508, and Lys607 are shown as a stick model and colored red. Other mutated residues (Glu501, Asp503, Leu504, Arg505, Asn506, Asn509, His534, Asp535, Phe569, Thr574, Asp576, Asn582, Gln584, Gln585, Asn586, Leu587, Asn588, Glu609) are shown as a stick model and colored orange. HA1, salmon and yellow; HA2, magenta; HA3, cyan; C-terminal region from Leu473 of HA3, pale cyan. (F) E-cadherin binding of HA/B and those containing HA3 point mutants in which each of the amino acid residues indicated in E was substituted with alanine was assessed by HA pull-down assay. The results show that Leu473, Met508, and Lys607 are critically involved in E-cadherin binding. (G) Caco-2 cells were grown on Transwell chambers, and treated with 10 nM HA/B complex or those harboring E-cadherin-binding-defective mutations (Leu473 to Ala, L473A; Met508 to Ala, M508A; Lys607 to Ala, K607A) from the lower side of the chambers. Values are means ± S.E.M. of triplicate wells. (H) Mucin binding of the HA/B complex or those harboring E-cadherin-binding-defective mutations. These mutations did not affect the carbohydrate binding. Values are means ± S.E.M. of three independent experiments. NS, not significant.

We previously showed that HA/A and HA/B interact with E-cadherin, but HA/C does not [15], [23]. By comparing HA/B and HA/C, we sought to identify the E-cadherin binding site. First, we constructed chimera HA complexes in which each HA subcomponent is replaced between HA/B and HA/C. E-cadherin binding was assessed by pull-down assay. As shown in Fig. 4A, all the chimeras that contained HA3/B interacted with E-cadherin, indicating that HA3 is the determinant of E-cadherin binding. Next, we constructed chimera HA3 between HA/B and HA/C to narrow down the determinant region further (Fig. 4B). HA3 is divided into four domains termed I, II, III, and IV [18]. We found that the C-terminal part of HA3/B from residue 473, which encompasses a C-terminal small portion of domain III and the entirety of domain IV, is required for E-cadherin binding (Fig. 4C). The binding was lost when the HA3/B region was further narrowed down to the C-terminal part from residue 485. Between residues 473 and 484, only two residues, Leu473 and Asn475, differ between HA3/B and HA3/C. Thus, we substituted each of these two residues in HA3/B to that of HA3/C, and examined E-cadherin binding. In a pull-down assay, mutation in Leu473 abolished the binding, whereas that in Asn475 did not affect it (Fig. 4D). Thus, Leu473 was found to be involved in E-cadherin binding.

To identify further the residues involved in E-cadherin binding, we selected the residues of HA3/B that fulfill the following criteria: residues located within the C-terminal part from Leu473, those differing between types B and C, and those located at the molecular surface adjacent to Leu473 (Fig. 4E). We mutated each of these residues to alanine, and E-cadherin binding was assessed by pull-down assay. As a result, binding was remarkably abrogated by mutating Met508 and Lys607 in addition to Leu473 (Fig. 4F).

Finally, we examined the influence of E-cadherin-binding-defective mutations on the barrier-disrupting activity by TER measurement. As shown in Fig. 4G, HA complexes containing each of these mutations in HA3 showed reduced activity compared with the wild-type complex. Remarkably, the complex containing Lys607Ala mutation completely lost its activity. Reduction in TER was not observed even when cells were treated with 100 nM of the mutant (data not shown). Carbohydrate binding activity was not reduced by this mutation (Fig. 4H). These results support our previous finding that the barrier-disrupting activity is completely dependent on E-cadherin binding [15].

## Discussion

Botulism often arises as a food-borne disease; however, BoNT alone is not sufficient to cause it and NTNH and HA are required for oral toxicity. In the case of serotype B, NTNH potentiates the oral toxicity of BoNT by about twenty-fold compared with BoNT alone. HA further potentiates the oral toxicity of BoNT. The oral toxicity of type B 16S toxin, which consists of BoNT, NTNH, and HA, is about seven hundred times higher than that of 12S toxin, which is comprised of BoNT and NTNH [8]. Thus, HA is crucial for oral poisoning and renders the toxin complex a unique oral toxin.

**Figure 5:**
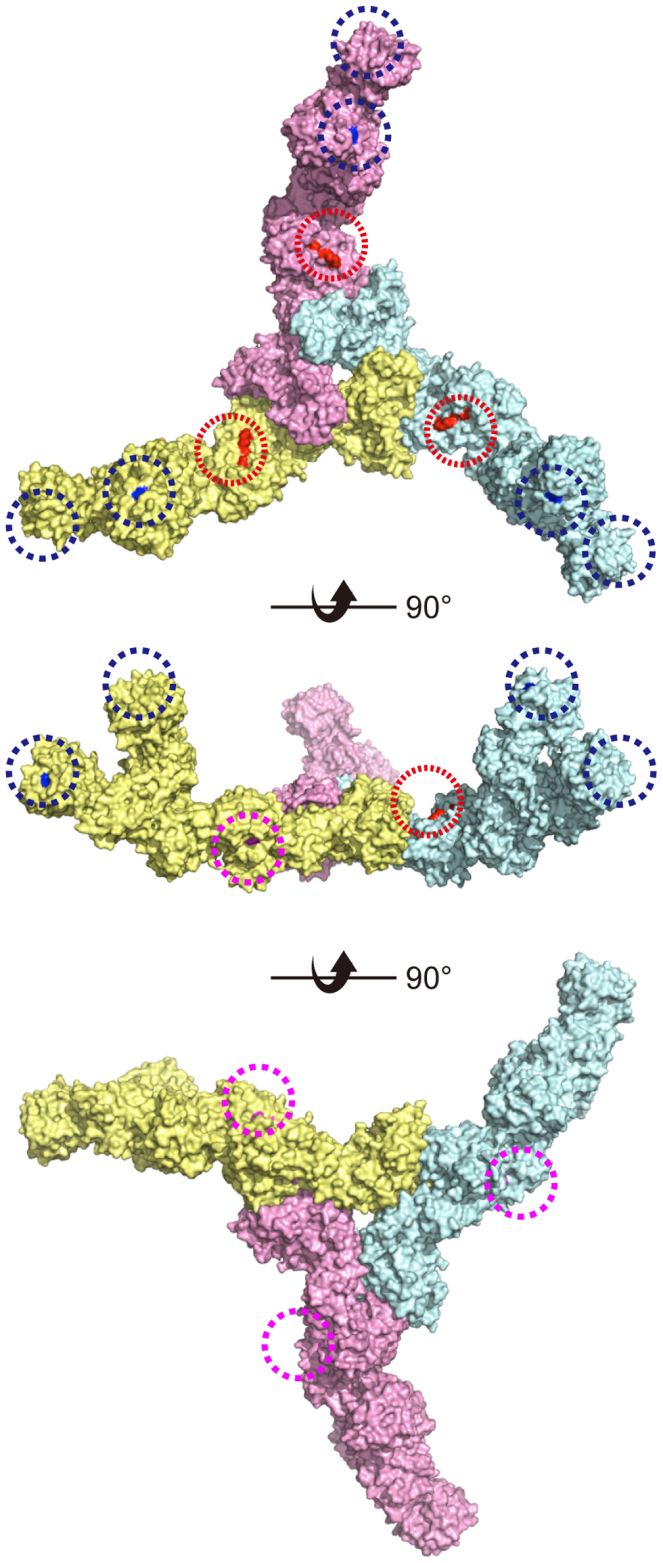
Crystal structure and the carbohydrate and E-cadherin binding sites of the HA/B complex. The galactose binding sites of HA1 (Asn286) are colored blue and marked with dotted blue circles. The sialic acid-binding sites of HA3 (Arg528) are colored magenta and marked with dotted magenta circles. The E-cadherin binding sites of HA3 (Leu473, M508, and Lys607) are colored red and marked with dotted red circles. These sites are structurally independent. Each of the three HA monomers is colored pale cyan, pale yellow, or pink.

HA possesses two specific activities, namely, carbohydrate binding and epithelial barrier disruption, the latter of which is dependent on E-cadherin binding [16]. It has been noted that carbohydrate binding of 16S toxin is attributable mainly to that of HA1 [12], [19]. This notion is readily understandable from the structures of HA complex and 16S toxin; the galactose binding sites of HA1 are positioned at the outer most parts of the complexes (Fig. 5) [10], [13]. The carbohydrate binding activity of HA1 was also prevalent in our experimental settings. In mucin binding assay, both BSM and PGM binding of the HA complex was greatly affected by galactose-binding-defective mutation of HA1. The inhibition of BSM binding by the HA1 mutation is apparently inconsistent with the result that GST-HA1 did not bind to BSM. Although BSM is rich in sialic acids, it also contains other carbohydrates including galactose; therefore, it is probable that HA1 proteins that constitute the HA complex recognize galactose moieties in BSM. There are three possible reasons why BSM binding of the HA complex was greatly inhibited by HA1 mutation, even though GST-HA1 did not bind to BSM. First, HA1 binding to BSM could be facilitated by sialic acid binding of HA3 present in the same complex. The observation that BSM binding of HA complex was greatly affected also by the sialic acid-binding-defective mutation of HA3 supports this notion (Fig. 1C). Second, HA1 binding to BSM could be enhanced in the HA complex due to multivalency effects. GST-HA1 is considered to behave as a divalent molecule due to dimerization through GST, whereas six galactose binding sites are present in the HA complex. We detected mucin binding of GST-HA1 but not Strep- or FLAG-tagged HA1, indicating profound effects of multivalency in carbohydrate binding (data not shown). Last, as described above, HA1 is positioned at the outer most part of the HA complex, in which the galactose binding sites are arranged in almost the same orientation, enabling cooperative ligand binding of these sites (Fig. 5), while this might not be the case in GST-HA1. Galactose binding of HA1 potentiated the barrier-disrupting activity by promoting cell surface binding and, in the case when HA was applied from the apical surface of cell monolayer, apical to basolateral translocation. In contrast to the galactose binding sites of HA1, sialic acid binding sites of HA3 are located at the inner part of the complex (Fig. 5) [10], [13]. Since this sialic acid binding site of HA3 is conserved among serotypes, it appears to have an important role in the pathogenesis of botulism. As shown in Fig. 1C, it is apparent that the sialic acid binding of HA3 renders the HA complex a more potent lectin with dual carbohydrate binding specificity. Meanwhile, although the sialic acid binding of HA3 enhances the barrier-disrupting activity, this enhancing effect was subtle and much more limited than that of galactose binding of HA1. It remains to be elucidated whether the sialic acid binding plays a critical role in the pathogenesis of botulism.

In this study, we identified E-cadherin binding sites in HA/B complex. We identified the critical residues by comparing HA/B and HA/C, the latter of which is unable to bind to E-cadherin [15]. These are Leu473, Met508, and Lys607, among which Lys607 appears to be critically involved in the interaction. E-cadherin binding was reduced or abolished by mutating these residues to alanine. Meanwhile, carbohydrate binding was not largely affected by these mutations. Reciprocally, HA complex harboring carbohydrate-binding-defective mutations showed comparable E-cadherin binding to that of the wild type. Consistent with these results, the residues involved in E-cadherin binding are located on the HA3 molecular surface that is opposite the sialic acid binding site of HA3 (Fig. 5). Collectively, we concluded that E-cadherin binding is structurally and functionally independent of carbohydrate binding.

We previously reported that HA/A also binds to E-cadherin and disrupts the epithelial barrier [15], [23]. The amino acid sequence identity between HA3/A (strain 62A) and HA3/B (strain Okra) is 98%, and the three residues that we identified as E-cadherin binding sites are completely conserved in HA3/A. While we were preparing this manuscript, the co-crystal structures of two distal extracellular cadherin domains of E-cadherin and a part of HA/A that includes HA1, HA2, and the C-terminal two domains, domains III and IV, of HA3 were solved [30]. The authors showed that this distal part of E-cadherin recognizes a molecular surface formed by HA2 and HA3, in which Leu473, Met508, and Lys607 are involved. This result is consistent with our previous notion that the HA2-3 connecting region of HA/B appears to be the E-cadherin binding site [18]. Thus, it is likely that HA/B binds to E-cadherin in the same manner as HA/A.

In conclusion, we identified carbohydrate and E-cadherin interaction sites in HA/B complex and showed that these two binding activities are functionally and structurally independent. Our result and the mutant constructs that are deficient in each of these functions would be useful to examine the roles of HA, especially those of sialic acid binding of HA3 and E-cadherin binding, in the pathogenesis of type B botulism.
